# Flat lens effect on seismic waves propagation in the subsoil

**DOI:** 10.1038/s41598-017-17661-y

**Published:** 2017-12-22

**Authors:** Stéphane Brûlé, Emmanuel H. Javelaud, Stefan Enoch, Sébastien Guenneau

**Affiliations:** 1 Ménard, 91620 Nozay, France; 2 Aix-Marseille Université, CNRS, Centrale Marseille, Institut Fresnel, 13013 Marseille, France

## Abstract

We show that seismic energy simulated by an artificial source that mainly propagates Rayleigh surface waves, is focused in structured soil made of a grid of holes distributed in the ground. We carry out large-scale field tests with a structured soil made of a grid consisting of cylindrical and vertical holes in the ground and a low frequency artificial source (<10 Hz). This allows the identification of a distribution of energy inside the grid, which can be interpreted as the consequence of a dynamic anisotropy akin to an effective negative refraction index. Such a flat lens reminiscent of what Veselago and Pendry envisioned for light opens avenues in seismic metamaterials to counteract partially or totally the most devastating components of seismic signals.

## Introduction

The concepts of photonic crystals^1,2^ and metamaterials^3^, emerged from nano-scale world and electromagnetism. In 2001, the word “metamaterial” was coined by R. M. Walser, who gave the following definition: macroscopic composite having a manmade, three-dimensional, periodic cellular architecture designed to produce an optimized combination, not available in nature, of two or more responses to specific excitation^4^. Typically, the terminology metamaterial encompasses periodic arrangements of elements with size comparable^1,2^ or much smaller than the considered wavelength (typically hundreds of nanometers for optical wavelength) that acquire effective properties of materials with unusual properties or applications such as negative optical index^5,6^, or highly anisotropic materials such as hyperbolic metamaterials^7^ or invisibility cloaking devices^8^. The transition from the electromagnetic to acoustic waves was made possible, in particular, thanks to phononic crystals, which are artificial handcrafted structures^9^. In August 2012, a first full-scale experiment was realized with a non-sub wavelength 2D grid of vertical empty cylindrical holes placed nearby a 50 Hz source^10^. This showed the interest in pursuing research on the interaction of structured soil with seismic waves.

The concept of seismic invisibility cloaking device means, for seismic waves, that the considered object (buildings, district, etc.) apparently does not affect the wavefield. Indeed, the high density of deep foundation or ground reinforcement techniques in the urban area, leads researchers to believe there is a significant interaction of these buried structures with a certain component of the seismic signal. A promising way to cause a modification on the seismic disturbance is to create complete artificial anisotropy by implementing geometrical elements, full or empty, in the soil^10^. The physical process is the interference of waves (body or surface waves) scattered from surfaces or objects. The effects of anisotropy are reinforced by the local resonance of implemented elements; these could theoretically lead to an ideal subwavelength cloak detouring waves around a protected area. In these periodic or non-periodic media, the desired effects are total reflection (Bragg’s effect), band-gaps, wave-path control, attenuation by energy-dissipation, amongst others.

**Figure 1:**
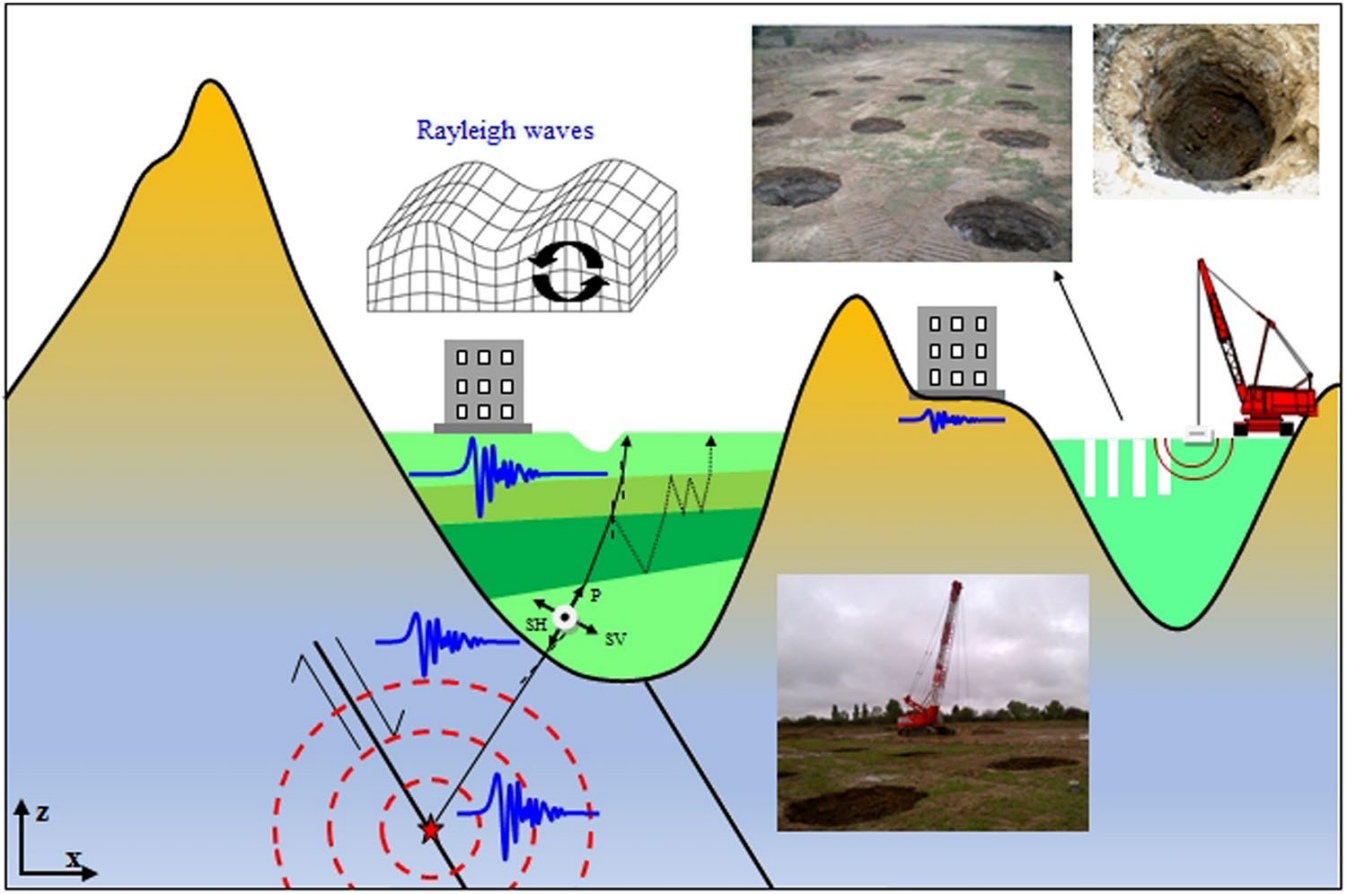
Illustration (cross-sectional view in the x–z-plane) of the principle of amplification of ground motion because of soft sediments covering rocky grounds, called “seismic site effect” (left) and seismic testing device (right) with photos of the periodically structured soil made of a 7.07 × 7.07 m square grid tilted through an angle of 45 degrees of 2 m diameter self-stable holes (23 units). As an artificial seismic source, the crane lifts and drops a weight of 17 tons on the surface. Each hole is preliminary created by the sole punching effect of the pounder (no cementation).

As a reminder we precise that structure damages due to seismic excitation are often directly correlated to local site condition (Fig. 1) in the form of motion amplification and/or soil liquefaction inducing ground deformation (see supplemental material for more details). This study is concerned with seismic signal compound of short wavelengths (tens to hundreds of meters) propagating and scattering in soft sediments basin. Figure 1, left part, shows the concept of verticalization of the seismic ray for pressure (P) and shear (S) waves inside basin of soft soil and the ground amplification effect. Let us consider a usual-type of focal mechanism as represented in Fig. 1. We point-out the fact that the geometrical spreading of seismic waves does not fully explain the ground motion recorded at the Earth’s surface. Indeed, because of a high contrast of impedance between rocky grounds and softer soils recovering them and also because of the wave-trapping phenomena and resonance, the seismic ground motion could be amplified.

We explore the low frequency range of vibrations by means of dynamic compaction sources. A test zone consisting of a regular mesh of vertical cylindrical voids was carried out near the city of Lyon (France) in September 2012.

The geology of the site is composed of alluvial and glacial deposits (sand, clay and pebbles). Thanks to a geological study^11^ describing a deep borehole (1 106 m) located at less than 500 m from the site (French Bureau de Recherches Géologiques et Minières) and presenting the 1D-plane parallel deposits, we know that the first strong seismic reflector is deeper than 400 m (coal schists). Lesser contrast impedances are observed at 70 m depth and especially at 170 m depth, due to denser conglomerate. Local *in situ* geotechnical tests, held especially for the worksite confirm that these hypotheses are legitimate at the scale of the experimental site.

**Figure 2:**
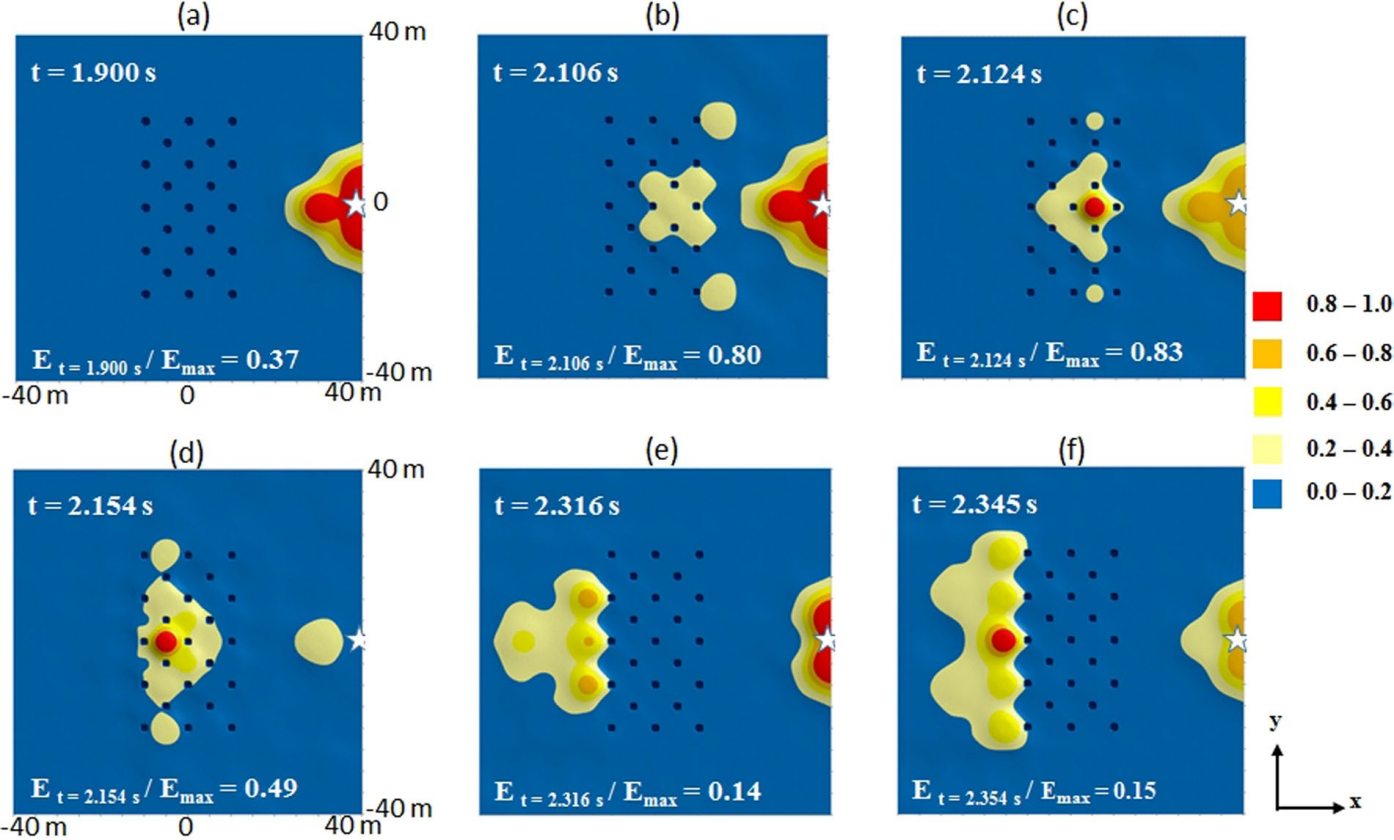
Sources S_1_ and S_6_, shot#6: chronology of the x–y spatial distribution of normalized *v* ^2^ *(t)* from 1.900 s (**a**) to 2.345 s (**f**). Source S_1_ (marked by a star) is located at (x = 40, y = 0), with coordinates in meters. Source S_6_ is located at (x = −40, y = 0). The impact is recorded at t = 1.85 s at sensor F5 located at 5 m from the source.

**Figure 3:**
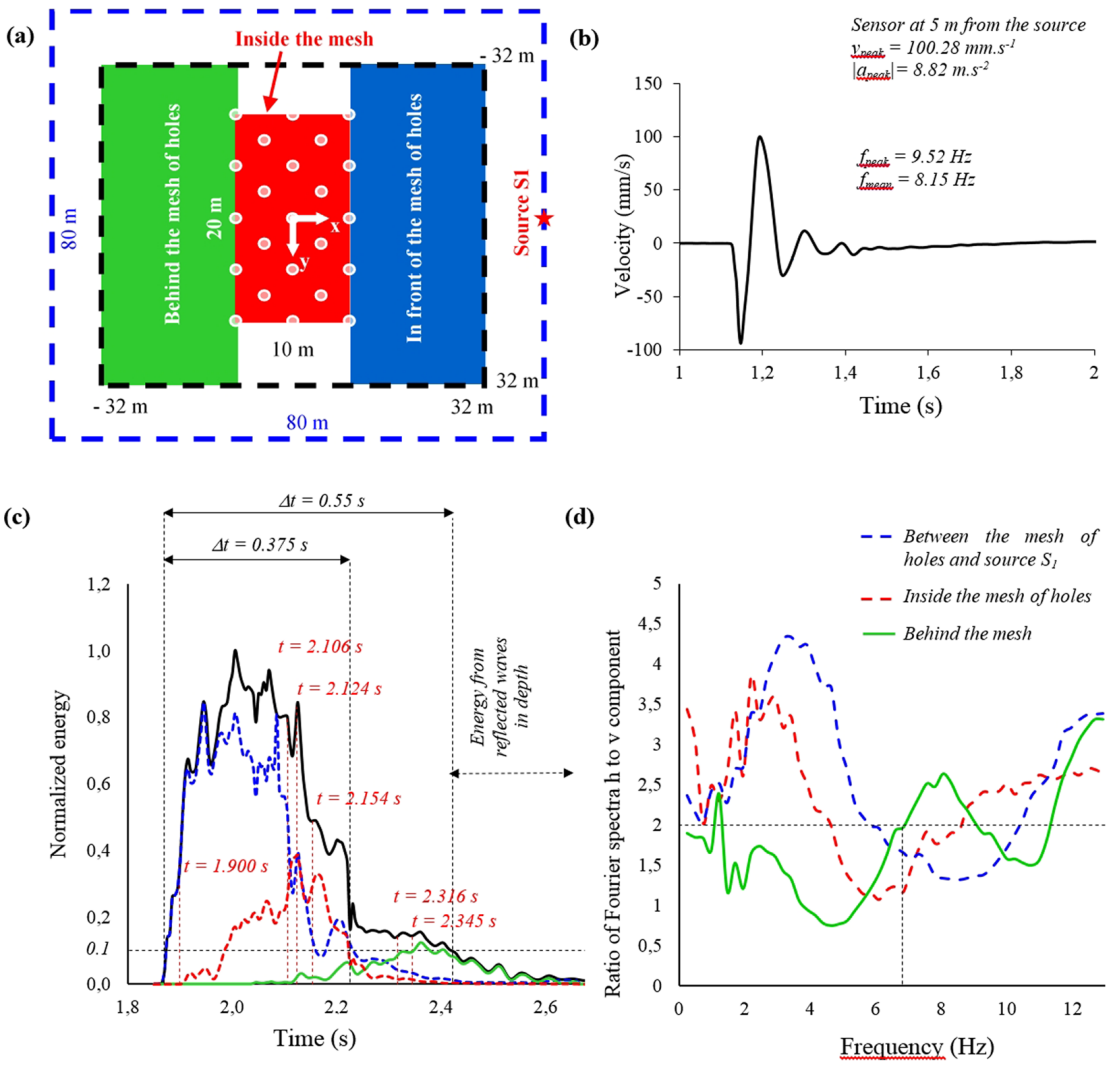
Localization of source S_1_ and the three areas of study: (**a**) in front, inside and behind the mesh of holes. Typical signal recorded in time domain (**b**) at 5 m from the source. Panel (c) shows the normalized mean energy *v* ^*2*^ *(t)* versus time for each surface depicted in (**a**). Above the black curve, we point the time of each sample illustrated in Fig. 2. Panel (d) represents the ratio of the horizontal over the vertical components of velocity versus frequency, defined as the ratio of Fourier spectra, computed for the three areas; for a given frequency, a value greater than 2, means a more important amplification of the horizontal component of the signal than the vertical one.

The experimental grid is made of 23 holes distributed along five discontinuous lines of self-stable boreholes 2 m in diameter (Figs 1, 2 and 3a, and Supplementary Figure 1). The depth of the boreholes is 5 m and the grid spacing is 7 m. The measurement of the velocity of the pressure wave in the soil is given by a preliminary seismic test, pointing the first wave arrival at various offsets from the source. We measured a velocity between 600 and 650 m/s for the earth material near the surface. Because of the geology, there is certainly a gradient of speed with the depth between two main seismic reflectors. The artificial source consists of the fall of a 17 ton steel pounder from a height of about 12 m to generate clear transient vibrations pulses (Fig. 3b). The typical waveform of the source in time-domain looks like a second order Ricker wavelet (or “Mexican hat wavelet”). However the trace shows a slightly increasing trend after the coda. This may be partly explained by the vicinity of the impact point and thus can be attributed to non-linear effects.

The signal is characterized by a mean frequency value at 8.15 Hz (λ_P-wave_~74 m) with a range of frequencies going from 3 to 20 Hz (30 < λ_P-wave_ < 200 m), *i.e*. this device is mainly “sub-wavelength” with hole’s spacing of 7 m in the horizontal plane. At 5 m from the impact, the peak ground acceleration is around 0.9 g (where g = 9.81 m.s^−2^ is the gravity of Earth), which is significant but necessary to compensate for the strong attenuation versus distance in earth materials. We precise that the vibration of the engine’s crane is very weak. The void grid spacing (7 m) is lower than the smallest wavelength measured. Because of the short distance between sources and sensors, we consider that body shear waves and Rayleigh waves almost arrive simultaneously. For the sake of information, making the assumption of an elastic soil, with a Poisson’s ratio between 0.3 and 0.40, it turns out that the ratio of pressure and shear wave speeds are respectively *v*_*p*_ */v*_*s*_ = *1.871* and 2*.449*. We then estimate the shear wave velocity to be around 250–300 m/s, what is coherent with the values known for this type of soil in earthquake engineering and associated correlations. Because of the geology of the site, i.e. a two-layer 1D-model of soil, made of a homogeneous soil lying on a deep substratum (depth: 70 or 170 m), we can calculate the theoretical fundamental frequency of the site for the first mode of vibration: F_0_ = V_s_/4 H. This parametric value of frequency is between 0.37 and 1.07 Hz.

Let us note that, a priori, the implementation of the void grid itself, may impact the properties of the initial ground and the time sample could contain direct and, due to local stratigraphy, reflected and refracted waves (Fig. 2); examining the traces of the seismograms, the amplitude of the refracted waves is very low. In the case of our experiment, the emitted signal is strongly polarized in the horizontal plane and more exactly in the x-direction i.e. perpendicularly to the side of the mesh (Supplementary Figure 2). This information is fundamental because it corroborates that most of the energy of the source is converted into energetic surface waves^12^, Rayleigh waves and perhaps also Love waves trapped between intermediate strata. We do not exclude the possibility of local resonance of S-H waves. First of all, this experiment is carried out to illustrate the interaction of a grid of holes with the horizontal component of the seismic motion.

To capture the ground motion’s field, we set 30 three-component velocimeters (*v*_*x*_ *, v*_*y*_ *, v*_*z*_) with a corner frequency of 4.5 Hz (−3 dB at 4.5 Hz) electronically corrected to 1 Hz. The sensors, were calibrated with other reference sensors (phase shifts, etc.) and were used simultaneously with a common time base and a delay of synchronization of +/−1 millisecond. The sensors were densely set in a quarter of the investigation area (Supplementary Figure 1). The pounder was consecutively dropped at five different places (sources location numbers 1, 2, 4, 6 & 7 in Supplementary Figure 1), and 7 to 12 times (acquisition 1 to 12) at each source location. Sensors remained fixed during the whole test and the complete field of velocity (80 m × 80 m) is obtained by means of the source symmetry (S_1_–S_6_) and the symmetry with respect to a plane passing through the x-axis (Supplementary Figure 1). Despite the vicinity of the first sensor near the impact (5 m) and the risk of non-stability, the waveform was correctly recorded.

## Results

The design of the grid of holes was defined by numerical simulation and parametric studies, using the Kirchhoff-Love plate theory dedicated to the analysis of flexural waves (see section Methods and Supplementary Information and Supplementary Figure 1). Results of the field test are presented in Figs 2 and 3. Sources S_1_ and S_6_ located at 30 m from the long side of the grid, simulate the case of a “far field” seismic signal. Sources S_2_ and S_7_, at 10 m, illustrate the “near-field” case (Figure S2). In Fig. 2, we present first a selection of pictures from the time history seismic test with sources S_1_ and S_6_ located at 30 m from the long side of the grid. The signal is unfiltered. We have only limited the high amplitude of the signal recorded by sensors located at 5 m from the impact point.

The impact is pointed at 1.85 s and we have selected 6 snapshots at t = 1.90, 2.106, 2.124, 2.154, 2.316 and 2.345 s. The duration of the shock is comprised between 0.3 to 0.4 s. In seismology the Husid’s diagram is often used to define a characteristic time duration *t*_*d*_ of an earthquake. Here, this value is 0.19 s, which is a rather short duration for real earthquake, where the S-wave window may have a tenfold duration.

An important step of this survey was to identify a representative physical quantity. We decided to select the normalized square velocity at each time step *v* ^2^ = *v*_*x*_^2^ + *v*_*y*_^2^ + *v*_*z*_^2^. Snapshots do not illustrate directly the propagation of the wavefront itself because we also observe, thanks to sensors located between the holes, a wealth of informations in the area located between the grid and the source, inside and behind the grid.

In time-domain, these pictures show two main phenomena. The first one is a significant wave reflection at the contact with the long-side of the grid up to 2.1 s. This phenomenon appears more markedly for the “near-field” case and it is characterized by strong concentration of energy in the back of the source (star-symbol). See Suplementary Figure S2 of Supplementary Information. The second phenomenon, illustrated here, is the transfer and transformation of the energy inside the grid of holes. One can observe a displacement of the concentration of energy inside the grid versus time (Fig. 3c) and more exactly, along the x-axis, with a succession of transient steady-states of the maxima. For the “near-field” experiment, due to the proximity of the source from the grid, the seismic signal arriving to the grid is less filtered in high frequency by soils, than in the case of the “far-field” test.

Figure 3c highligths the order of magnitude of the energy at each time step and permits to identify the origin of the signal (direct or backscattered). The total area is divided into three regions: inside (red line), behind (green line) and in front of the grid (blue line), as illustrated in Fig. 3a. For each region and for the whole surface (black line), we plot the sum of the quantity *v* ^2^ recorded for all sensors, at a given time. This value is normalized by the maximum recorded during all the time history. One can recognize the duration of the source signal (Δt = 0.375 s) and probably from time 2.2 s, the energy of the backscattering signal. We consider that energy fields presented in Fig. 2 at times t = 1.90, 2.106, 2.124 and 2.154 s are representative of the direct signal coming from the source. However, fields at t = 2.316 and 2.345 s could contain a part of different reflected waves due to the geological strata. In fact, assuming an existing reflector at 170 m of depth, and a velocity of 600 m/s, the first time arrivals of the reflected wave are 2.4 and 2.42 s respectively at the right and left long side of the grid. However the amplitude of scattered waves would be much weaker than the direct signal. To identify the effect of various reflected waves at different depths, which have dominant vertical components for first time arrival, due to the small acquisition offset, we also carried out the study of *v* ^2^ = *v*_*x*_^2^ + *v*_*y*_^2^. Results are similar.

In frequency-domain, we chose to make use of the technique inspired from “H/V” spectral ratio; it is portion of the horizontal-to-vertical spectral ratio (HVSR) seismic ambient-noise method, but it is not the technique itself which requires for example a certain duration of recording of the seismic noise and not an artificial source. The analysis of data^13,14^ consists in estimating the ratio between the Fourier amplitude spectra of the horizontal to vertical components of ambient noise vibrations recorded at each sensor. This procedure is used to obtain horizontal to vertical (H/V) spectral ratios from any type of vibration signals (ambient vibrations, earthquake…). We have calculated the ratio *(|T*_*f*_ *(v*_*x*_ *)|* + *|T*_*f*_ *(v*_*y*_ *)|)/|T*_*f*_ *(v*_*z*_ *)|*. At a given frequency, a value greater than 2, means a more important amplification of the horizontal component of the signal than the vertical one. In Fig. 3d, we present the mean value of this ratio for a given area versus frequency: in front (blue curve), inside (red curve) and behind (green curve) the grid. In the range of 1 to 12 Hz, the shape of these three curves is similar with a first portion of 2 to 4 Hz of width, where the spectral ratio is greater than two. In a portion of the chart, the spectral ratio is below 2. The difference between these three curves is the location of minima and maxima. The spectral ratio is greater than 2 from 1 to 5 Hz for the inside-grid area and lower than 2 from 5 to 9 Hz. For the data recorded at the front surface, we note a frequency right-shift (1.5 to 3 Hz) of the red curve to the blue one. The range of ratio values lower than 2 is between 1 and 7 Hz for the green curve (back surface). Beforehand we verified the ellipticity of Rayleigh waves does not affect the results (see Supplementary Information).

In principle, we should not excite the resonance of the basin due to frequency bandwidth of the source (3 to 20 Hz), but this hypothesis cannot be ruled out completely. Indeed, in Fig. 3d, one can note a peak of amplification between 1 and 2 Hz but this is not a “HVSR” analysis. However, we consider that the energy of the source is important but not large enough to excite S-waves of the whole basin.

## Discussion

In time-domain, the field tests show a focusing of seismic energy inside a mesh of empty holes with sub-wavelength grid spacing. This phenomenon may be interpreted as some form of negative refraction for surface seismic waves in a way similar to what was observed for pulse focusing of flexural waves in a 45 degree tilted square array of circular holes in a plate^15^. Here we show Veselago-Pendry like lensing for seismic waves. In frequency-domain, we point out the capability of the grid to accentuate the filtering of the horizontal components of the signal in the range of 2 to 7 Hz, in this case. This result is corroborated, in time and spectral domains, for back surface by means of a preliminary test carried out with a linear land-streamer (offset of sensors: 5, 10, 20, 50 and 70 m) on a soil with and without a hole.The origin of the phenomenon is the selective reflection in the front surface and probably complex changes of wave polarization at each interface. Although Rayleigh waves have more complex (elliptical) polarization than flexural waves in plates, it turns out that seismic metamaterials behave in many ways as platonic crystals which are periodically structured plates displaying some dynamic anisotropy allowing for lensing and highly directive emission effects. This survey on a 2D-seismic metamaterial highlights the reality of the interaction of buried structures with Rayleigh surface waves of short seismic wavelength (tens to hundreds of meters).

In this article we presented all the demanding but necessary conditions to carry out a full-scale experiment with structured holey-soils and artificial seismic source. Subject to somewhat restrictive hypotheses, we have shown that it is possible to act significantly on the propagation of seismic waves (distribution of energy, frequency content) passing through artificially structured soils, as foreseen in^16,17^. This opens an avenue for the research on the control of seismic waves via dynamic soil anisotropy. In the present case, the hyperbolic-type lensing effect for Rayleigh waves has been captured by an asymptotic model known as high-frequency homogenization for thin structured plates^18,19^. Interestingly, in the tracks of our first experiment on a seismic shield^10^, numerous studies appeared on large scale mechanical metamaterials with low frequency stop bands^20–26^. It is even possible to shield Rayleigh waves with pillars^27^ and trees^28^ periodically arranged atop elastic substrates. In the future, structured soils, among which metamaterials, could be complementary to the existing passive earthquake engineering techniques and, with the perspective to realize such devices under and/or around a building, the anisotropy could be obtained by means of 2D (mesh of concrete or steel columns) or 3D devices (cells made of walls). The case of S-waves with strictly vertical incidence to the surface remains to be explored but here we show the interest of interacting with the horizontal component of the seismic waves. We hope our work will foster further theoretical and experimental efforts towards a seismic cloak^29^ and other types of seismic metamaterials inspired by research in photonics and using currently available civil engineering techniques^30^.

## Methods

**Figure 4:**
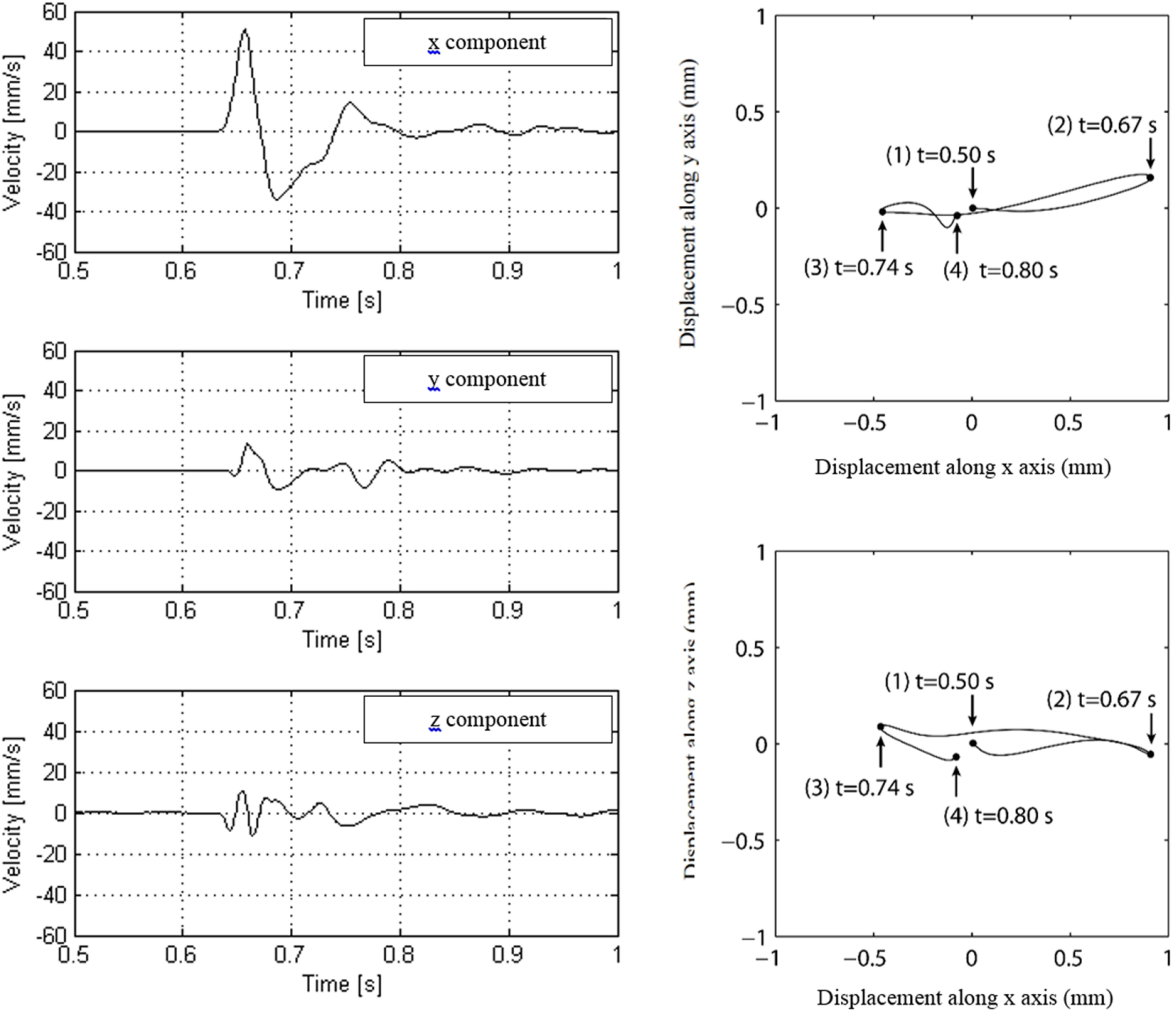
Left, velocity versus time recorded at 10 m from the pounder impact during the preliminary seismic tests (acquisition n°1). Right, displacement’s orbit (top, from above, i.e. y-component versus x-component and bottom, from the side, i.e. z-component versus x).

To facilitate the interpretation of results presented in this article, we first recall the particularity of Rayleigh waves in terms of ellipticity and we present the specific analysis of the ground motion polarization, thanks to data recorded at 10 m from the impact in Fig. 4. Finally, we propose an approximate plate model that qualitatively reproduces the observed effects.

### Ellipticity of Rayleigh waves

In the left part of Fig. 4, we present the velocity data (x, y, z) recorded at 10 m from the impact. In the right part, we show the related plots of particle motion in horizontal (x–y) plane and vertical (x–z) plane. In this case, the signal is strongly polarized in the horizontal plane and more exactly in the x-direction i.e. perpendicularly to the side of the mesh. This information is essential because it corroborates that most of the energy of the source is converted into energetic surface waves. This ellipticity may complicate the interpretation of results and the effectiveness of the grid of holes. As for velocity dispersion, the Rayleigh wave motion is frequency dependent and such an ellipticity function can be represented by the Fourier transform ratio of the horizontal and the vertical components of motion versus frequency. The shape of this function could be “flat”, if the velocity of compressional and shear waves for first superficial strata do not vary brutally versus depth at each interface. The preliminary seismic tests carried out on the virgin ground, confirm the flatness of this function in the range of 2 to 11 Hz, in accordance with the geological data.

### Numerical results

Let us now describe some numerical simulations, which provide some phenomenological understanding of wave phenomena at stake in the experiments led by the Ménard company. Much has been said about control of light, sound, water, or shear (SH) waves^31–36^ using the rich behavior encapsulated by the dispersion curves of bi-periodic structures, modeled by a Helmholtz equation ${\rm{\Delta }}u+{k}^{2}u=0$, with ${\rm{\Delta }}=\frac{{{\rm{\partial }}}^{2}}{{\rm{\partial }}{x}^{2}}+\frac{{{\rm{\partial }}}^{2}}{{\rm{\partial }}{y}^{2}}+\frac{{{\rm{\partial }}}^{2}}{{\rm{\partial }}{z}^{2}}$ the Laplacian, up to minor changes in the normalization of material parameters, and choice of boundary conditions (e.g. Dirichlet or Neumann for clamped or freely vibrating inclusions in the context of SH waves). However, when one considers elastic waves, governed by the vector Navier equations, which for an isotropic homogeneous medium take the following form in the frequency domain (1):

1 $$(\lambda +2\mu )\nabla (\nabla \cdot {\rm{U}})-\mu \nabla \times (\nabla \times {\rm{U}})=-{\rm{\rho }}{\omega }^{2}{\rm{U}}$$

with *ω* the angular wave frequency (in unit of rad.s^−1^), λ and µ the Lamé parameters (Pa), and ρ the density of the elastic medium (kg.m^−3^), things become more complicated. Indeed, the unknown *U*(*x*,*y*,*z*) is a 3-component displacement field, and $\nabla =(\frac{\partial }{\partial x},\frac{\partial }{\partial y},\frac{\partial }{\partial z})$ denotes the gradient with partial derivatives in all three coordinates, whereas . and × denote the usual scalar and cross products in Cartesian coordinates. When the elastic medium is homogeneous, one can still decouple the Navier equation(1) in Helmholtz-type equations, but if one considers stress-free inclusions in the elastic medium, it is no longer possible to reduce the analysis to scalar partial differential equations (PDEs), as shear and pressure waves do couple at inclusions’ boundaries. This makes difficult correspondences between electromagnetic and elastodynamic metamaterials.

There is nevertheless, the simplified framework of the Kirchhoff-Love plate theory^37^ that allows for bending moments and transverse shear forces to be taken into account via a fourth-order PDE for the out-of-plane plate displacement field. This plate theory is a natural extension of the Helmholtz equation to a generic model for flexural wave propagation through any spatially varying elastic medium thin compared with the typical wave wavelength. This PDE is deduced from an asymptotic analysis in (1), that amounts to taking a limit of small plate thickness in out-of-plane coordinate z, which leads to^37,38^

2 $$D{{\rm{\Delta }}}^{2}u-{\rm{\rho }}h{\omega }^{2}{\rm{u}}=0$$

where D = Eh^3^/(12(1 − *ν* ^2^)) is the so-called plate flexural rigidity, h its thickness, E the Young’s modulus and *ν* the Poisson ratio, which are such that E = λ(1 + *ν*)(1 − 2*ν*)/*ν*. Moreover, u is the third component of U.

Kirchhoff-Love equation(2) offers a very convenient mathematical model to draw analogies between the physics of electromagnetic and mechanical metamaterials. One can then transfer earlier knowledge gained in photonic or platonic crystals to large scale seismic metamaterials. However, while the Helmholtz equation can, with appropriate notational and linguistic changes, hold for acoustic, electromagnetic, water or out-of-plane elastic waves and so encompasses many possible applications, the Kirchhoff-Love plate theory is dedicated to the analysis of flexural waves (it does not model propagation of in-plane elastic waves in platonic crystals). Nonetheless, (2) can be used as a simplified, but meaningful, model for Rayleigh waves in structured soils^10^. Correspondences between models for scalar waves in photonic crystals and surface elastic waves in structured soils are unveiled when one recasts Eq. (2) as per:

3 $$(\frac{{\partial }^{2}}{\partial {x}^{2}}+\frac{{\partial }^{2}}{\partial {y}^{2}}+{\rm{\Omega }})(\frac{{\partial }^{2}}{\partial {x}^{2}}+\frac{{\partial }^{2}}{\partial {y}^{2}}-{\rm{\Omega }})u=0$$

where ${{\rm{\Omega }}}^{2}=\frac{12(1-{\nu }^{2})\rho {\omega }^{2}}{E{h}^{2}}$ Upon inspection, (3) looks like a factor of two Helmholtz equations with spectral parameters k^2^ = Ω and (ik)^2^ = Ω with i^2^ = −1. Physically this means that one has coexistence of propagating and evanescent flexural waves in the plate.

To model the soil structured with boreholes, we consider a plate with same geometric and soil parameters as in the experiments and with thickness 5 m, which contains an array of 23 stress-free inclusions. This is an approximate model where the moment and the transverse force are both equal to zero. This means we set the following two boundary conditions on each air hole (written in polar coordinates for simplicity as we consider circular holes):

4 $$D\frac{{\partial }^{2}u+}{\partial {r}^{2}}+Dv(\frac{1\partial u}{r\partial r}+\frac{{\partial }^{2}u}{{r}^{2}\partial {\theta }^{2}})=0$$

5 $$D\frac{\partial }{\partial r}(\frac{{\partial }^{2}u}{\partial {r}^{2}}+\frac{1\partial u}{r\partial r}+\frac{{\partial }^{2}u}{{r}^{2}\partial {\theta }^{2}})+\frac{1}{r}D(1-v)\frac{\partial }{\partial \theta }(\frac{1}{r}\frac{{\partial }^{2}u}{\partial r\partial \theta }-\frac{{\partial }^{2}u}{{r}^{2}\partial {\theta }^{}})=0$$

where (4) enforces vanishing moment and (5) enforces vanishing transverse force.

**Figure 5:**
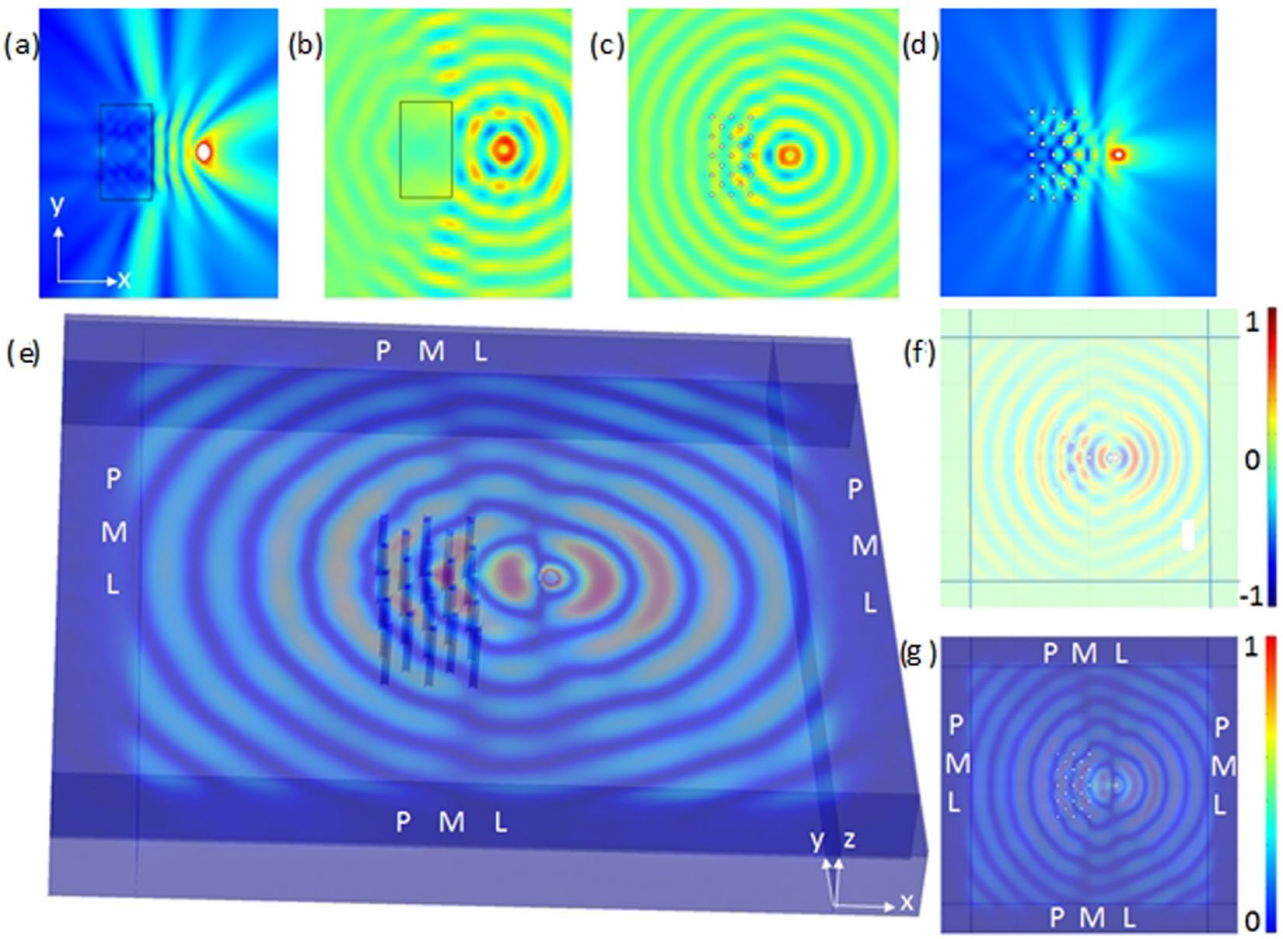
Numerical simulations showing lensing effect in a thin plate of thickness 5 m for a time-harmonic point source at 8 Hz placed near a flat lens with effective parameters T_11_ = 14, T_22_ = −10 with 2D plots of Re(u) (**a**) and |u| (**b**) solutions of Eq.(2) to be compared with same for freely vibrating inclusions in (**c**) and (**d**). Lensing effect can be also observed in 3D plot of the modulus of the three component vector field |U| (**e**), and on 2D plots of Re(U_3_) (**f**) and |U_3_| (**g**) in the xy-plane by solving the full elastic Navier system Eq.(1)

It is possible to show using high-frequency homogenization^21^ that the solution u satisfies the following effective dispersion relation ${\rm{\Omega }}\sim {{\rm{\Omega }}}_{0}-{T}_{ij}/(2{{\rm{\Omega }}}_{0}){\kappa }_{i}{\kappa }_{j}$ where T_ij_ is a rank-2 tensor encompassing the (frequency dependent) effective elastic parameters, κ_i_ is a component of the Bloch wavenumber and Ω_0_ is the standing wave frequency at the Brillouin zone edge 0, −π/2, π/2 depending on the location in the Brillouin zone about which the asymptotic expansion originates. For symmetry reasons, only diagonal components *T*_*ii*_ are non- zero. Besides from that, when T_11_T_22_ < 0 the effective dispersion relation describes some hyperbolic type wave propagation i.e. waves propagate like in the geometrical ray optics theory^19^, and this happens notably at a frequency around 8 Hz, see Fig. 5a and b, where a point source gives rise to an image inside and a faded one outside a flat slab with effective parameters T_11_ = 31 and T_22_ = −14 whereas T_11_ = T_22_ = 1 outside the slab, in accordance with same simulation for the array of air holes, see Fig. 5c and d for plots of Re(u) and |u|, respectively. Note that elastic parameters of soil are embedded in Ω for which a Young modulus E = 0.153 GPa, a Poisson ratio *ν* = 0.3 and a density ρ = 1800 kg m^−3^ are considered, as well as a plate thickness h = 5 m. This theoretical prediction for a hyperbolic-type metamaterial is in accordance with Fig. 3d, where both the red and green curves display a local maximum around 8 Hz, which coincides with a minimum for blue curve. On the other hand, when |T_11_| << |T_22_|, one observes some cross like wave propagation, which happens at frequency around 3 Hz, and is akin to the endoscope effect discussed in^19^. We have checked that these theoretical predictions still hold for the full elasticity case by solving the full Navier equations (1) for a plate with thickness 15 m, with stress free boundary conditions on cylindrical inclusions of diameter 2 m and height 15 m (considering cylindrical inclusions of height 5 m does not alter the plots at 8 Hz), and all other boundaries of the domain. Unwanted reflections on all lateral sides of the computational domain are avoided using 3D perfectly matched boundary layers implemented as described in^25^. One can clearly see the lensing effect on the 3D plot of the modulus of the vector displacement field |U| (Fig. 5e), as well as on 2D plots of a slice of the real part Re(U_3_) (Fig. 5f) and modulus |U_3_| (Fig. 5g) of its vertical component taken close to the upper boundary of the plate. Note that we consider in 3D some plate of thickness 15 m to make sure we generate ‘Rayleigh-like’ waves and not Lamb waves anymore like in the 2D flexural wave case, see^25^ for similar computations for the case of stiff inclusions in soil clamped to a bedrock.

As one can see, this dynamic effective anisotropy is essential in seismic metamaterials as it enables to mould the flow of surface elastic waves almost at will, as was theoretically shown in the case of thin plates^19^, with an experimental proof of concept for an acoustic source generating hyperbolic-type Lamb wave patterns in a periodically pinned plate at kHz frequencies^39^. Importantly, homogenized models of thin and thick plates have much in common, and the former provide useful guidance for the latter^26^. For instance, analogies between models of thin and thick plates makes possible the design of seismic cloaks via artificial anisotropy^25,40^.

## Electronic supplementary material

Supplementary Information
