# Supplementary material for: Flat lens effect on seismic waves propagation in the subsoil
Source: Sci Rep. 2017 Dec 22;7:18066. doi: 10.1038/s41598-017-17661-y (PMC5741748; doi:10.1038/s41598-017-17661-y)
Supplement: Supplementary file 1 — Supplementary Information [file 41598_2017_17661_MOESM1_ESM.pdf]

Supplementary Information for

Flat lens effect on seismic waves propagation in the subsoil

S. Brûlé, E.H. Javelaud, S. Enoch, S. Guenneau.

correspondence to: stephane.brule@menard-mail.fr , sebastien.guenneau@fresnel.fr.

We first recall in section S1 what is a seismic site effect, which is a major concept in seismology and earthquake engineering, in particular, to identify the range of applicability of seismic metamaterials. Section S2 is an overview of the field tests depicted in Supplementary Figure S1 (holes, sensors, sources). Finally, in section S3 we describe the additional results obtained during the "near-field" tests, with a source located at 10 m from the long side of the grid of holes (Supplementary Figure S2).

S1. Seismic site effect

When seismic waves propagate through sediment layers (Figure 1 of main article) or scatter on strong topographic irregularities, refraction/scattering phenomena may strongly increase the amplitude and the duration of the ground motion. Body waves (compressional and shear waves) propagate from the seismic source to the Earth surface. For surface waves, it is of foremost importance to distinguish long period surface waves (low frequency, i.e. < 1 Hz) travelling along the Earth's surface, on the crust, and short ones (< 10 Hz) mainly generated in case of site effects described above or by human activities at the Earth surface. A fundamental fact is the low value of surface wave velocity, generated by natural seismic source or construction work activities, in superficial and under-consolidated recent subgrade: less than 100 m/s to 400 m/s for shear wave. In these soil layers, considering the 0.1 to 10 Hz frequency range, wavelengths of induced surface waves are shorter than that of direct P and S waves: from a few meters to hundreds of meters. This order of wavelength is similar to those of buildings. This is the reason why we can expect building's resonance phenomena to occur with some soil in case of earthquakes (such as in the 2009 L'Aquila earthquake in Italy or more recently, in the 2015 Kathmandu earthquake in Nepal and the three 2016 earthquakes that struck Italy again on the 22nd of August, the 26th of October and the 30th of October, about 100 km north-east of Rome, not far from L'Aquila), which makes it possible to conceive seismic metamaterials whose size could be similar to that of the building project.

S2. Geometrical characteristics of the device

In Supplementary Figure S1, we present an overview of the field-device. The experimental grid is made of 23 holes distributed on five discontinuous lines of self-stable boreholes 2 m in diameter. The depth of the boreholes is 5 m and the grid spacing is 7.07 m. The spacing between sensors is 10 m in vertical and horizontal direction, except in the grid of holes where the vertical spacing is 5 m. The source is located at 10 m ("relative near-field" configuration $S_1 – S_6$) or at 30 m ("relative far field" configuration $S_2 – S_7$) from the long side of the grid.

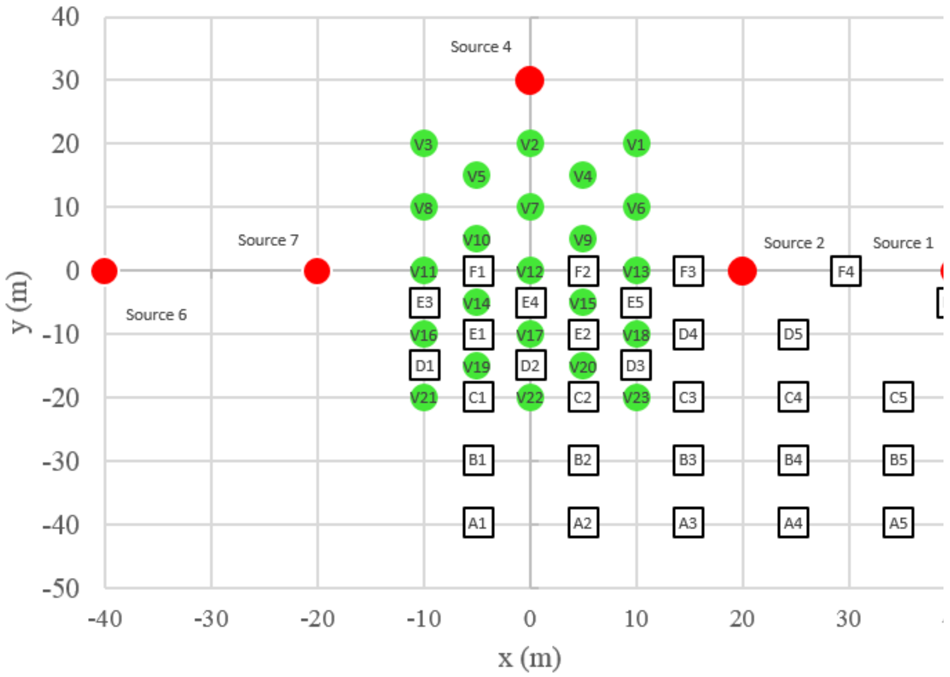

Supplementary Figure S1. Overview of the field tests: grid of holes (green disks), mesh of sensors (black rectangles) and sources' positions (red disks).

S3. "Near-field" results

In Supplementary Figure S2, we present a selection of snapshots illustrating the field of v^2, sampled from the time history of the seismic test. Sources are S_2 and S_7, located at 10 m from the long side of the grid. The impact is pointed at 1.85 s and we have selected 4 pictures at t = 1.887, 1.909, 2.069 and 2.227 s. These pictures are selected to illustrate the strong reflection of the signal during all the duration of the impact (around 0.3 s), with two refocalization effects: one inside the grid around t = 2.069 s and a second one around t = 2.227 s. Due to the proximity of the source from the grid, the seismic signal arriving at the grid is less filtered in high frequency by soils, than in the case of the "far-field" tests.

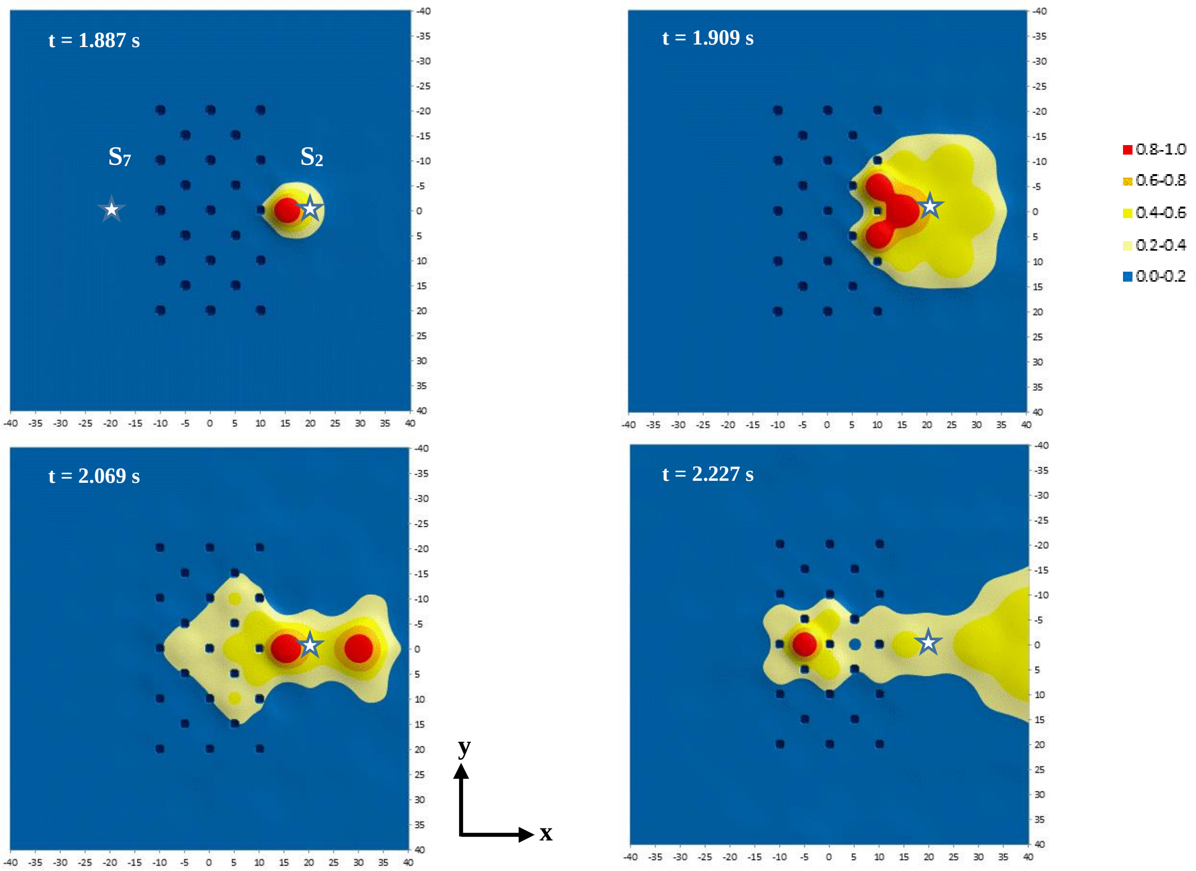

Supplementary Figure S2. Sources S_2 and S_7, shot#3: chronology of the x-y spatial distribution of normalized $v^2(t)$ from 1.887 to 2.227 s. Source S_2 is located at (x=20, y=0), with coordinates in meters. Source S_7 is located at (x=-20, y=0). The impact (marked by a star) is recorded at t=1.87 s at sensor F3 which is located at 5 m from the source.
